# Room-temperature Operation of Low-voltage, Non-volatile, Compound-semiconductor Memory Cells

**DOI:** 10.1038/s41598-019-45370-1

**Published:** 2019-06-20

**Authors:** Ofogh Tizno, Andrew R. J. Marshall, Natalia Fernández-Delgado, Miriam Herrera, Sergio I. Molina, Manus Hayne

**Affiliations:** 1 Department of Physics, Lancaster University, Lancaster, LA1 4YB UK; 2 Department of Material Science, Metallurgical Engineering and Inorganic Chemistry, IMEYMAT, University of Cádiz, 11510 Puerto Real, Cádiz Spain

**Keywords:** Electrical and electronic engineering, Semiconductors, Electronics, photonics and device physics

## Abstract

Whilst the different forms of conventional (charge-based) memories are well suited to their individual roles in computers and other electronic devices, flaws in their properties mean that intensive research into alternative, or emerging, memories continues. In particular, the goal of simultaneously achieving the contradictory requirements of non-volatility and fast, low-voltage (low-energy) switching has proved challenging. Here, we report an oxide-free, floating-gate memory cell based on III-V semiconductor heterostructures with a junctionless channel and non-destructive read of the stored data. Non-volatile data retention of at least 10^4^ s in combination with switching at ≤2.6 V is achieved by use of the extraordinary 2.1 eV conduction band offsets of InAs/AlSb and a triple-barrier resonant tunnelling structure. The combination of low-voltage operation and small capacitance implies intrinsic switching energy per unit area that is 100 and 1000 times smaller than dynamic random access memory and Flash respectively. The device may thus be considered as a new emerging memory with considerable potential.

## Introduction

Static random access memory (SRAM), dynamic random access memory (DRAM) and Flash have complementary characteristics that make them well-suited to their specialised roles in cache, active memory and data storage, respectively. Nevertheless, each of them have their drawbacks. Flash memories, first introduced in 1984, are essentially metal-oxide-semiconductor field-effect transistors (MOSFETs) with an additional floating gate (FG) for charge storage^1^. Data are represented by the quantity of charge held in the FG, which is isolated by oxide layers. However, the robust charge storage required for non-volatility comes at a cost. Writing and erasing requires application of a large voltage to the control gate (CG), typically ±20 V^2^. The process is slow, and induces voltage-accelerated failure mechanisms in the oxide^3,4^, limiting the endurance of the device. On the other hand, only small voltages are needed to read the data by testing the conductivity of the channel. This is efficient, and leaves the data intact, which is known as non-destructive read. In contrast to Flash, all DRAM single bit operations are relatively fast, making it the workhorse of active memory. However, data is lost from DRAM cells when it is read^5^. Furthermore, charge leaks from the capacitors used to store the data, so DRAM also has to be refreshed every few tens of ms. SRAM is the fastest conventional memory, and has relatively good data retention compared with DRAM, but typically uses six transistors per cell, and so requires a large footprint on the chip. These issues mean that despite the evident long-standing success of conventional memories, the search for alternatives, so-called emerging memories, continues unabated^6,7^. Charge trap memory^8,9^, phase change memory^10,11^, ferroelectric RAM^12,13^, resistive RAM^14,15^, conductive bridge RAM^16,17^ and magnetoresistive RAM^18,19^, collectively called storage-class memory (SCM)^20^ are all examples of emerging memories which have been subject to vigorous research activity.

Here we report on the conception^21^, design, modelling, fabrication and room-temperature operation of a novel, low-voltage, compound-semiconductor, charge-based, non-volatile memory device with compact form. Exploitation of the spectacular conduction-band line-up of AlSb/InAs for charge retention, and for the formation of a resonant-tunnelling barrier, has enabled us to demonstrate the contradictory characteristics of low-voltage (low-energy) operation and non-volatile storage^7^. The device is a FG memory structure made of InAs/AlSb/GaSb heterostructures, with InAs used as both FG and the junctionless channel. Simulations are undertaken to demonstrate the device operation concept, while the key memory properties of our device, such as the retention characteristics of the programmed/erased states, are presented as experimental results on fully-operational single cell devices.

### Operation concept

**Figure 1:**
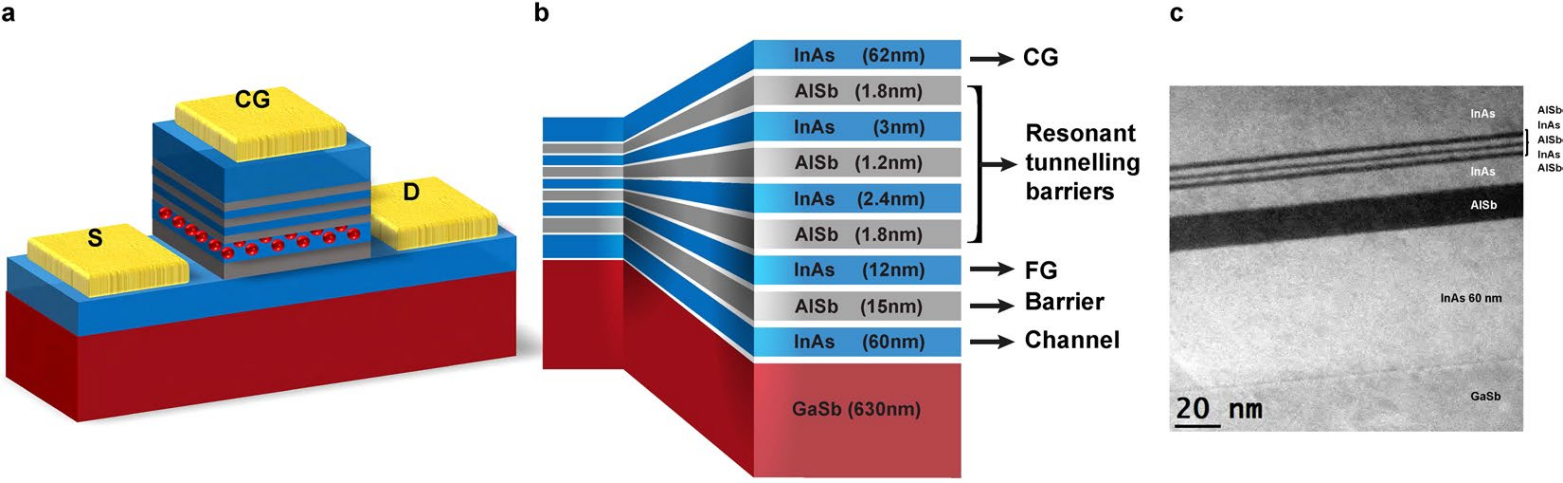
Device structure. (**a**) Schematic of the processed device with control gate (CG), source (S) and drain (D) contacts (gold). The red spheres represent stored charge in the floating gate (FG). (**b**) Details of the layer structure within the device. In both (**a**,**b**) InAs is coloured blue, AlSb grey and GaSb dark red. (**c**) Cross-sectional scanning transmission electron microscopy image showing the high quality of the epitaxial material, the individual layers and their heterointerfaces.

**Figure 2:**
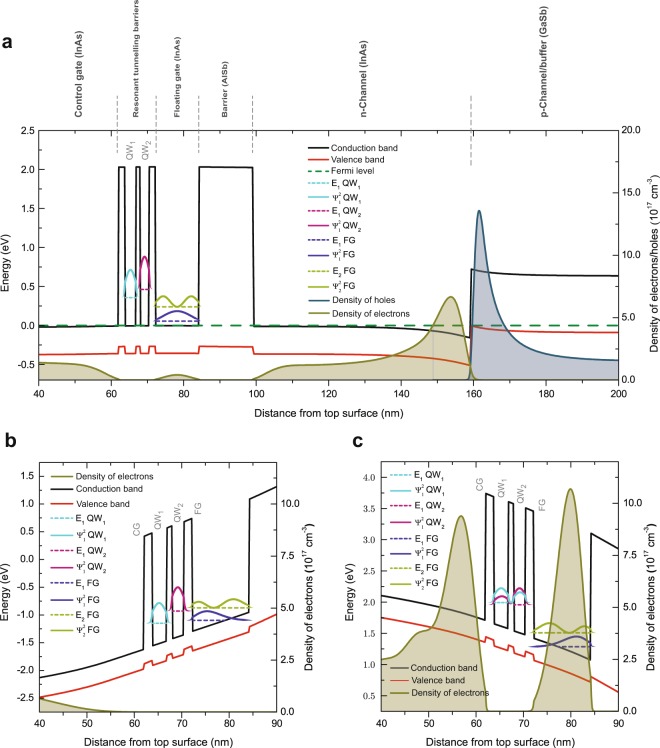
Calculated band diagram vertically through the structure. The black and red lines represent the bottom of the conduction band and the top of the valence band respectively, whilst the green long-dashed line is the Fermi-level. Short-dashed lines represent energies of the lowest confined states in the quantum wells (QWs) of the resonant-tunnelling structure, and the ground and the first excited-states in the floating gate (FG). The probability densities, ${|{\rm{\Psi }}|}^{2}$, for the position of the electrons in the QWs and the FG are plotted in arbitrary units. The densities of electrons and holes are plotted as olive and blue shaded areas respectively. (**a**) In the absence of bias across the device. (**b**) At the end of the erase process with ${V}_{CG-S}^{E}$ = +2.6 V, showing the depletion of the FG of electrons. (**c**) At the end of the write process when ${V}_{CG-S}^{W}$ = −2.6 V. This results in a significant density of electrons in the FG.

Figure 1 is a schematic representation of the memory cell, along with a cross-sectional, high-angle, annular, dark-field, scanning transmission electron microscopy (HAADF-STEM) image of the epitaxially-grown material used in this work. Like Flash, charge is stored in a FG. However, there are no oxide barriers. Instead, we have exploited the conduction band offsets in the so-called 6.1-Å family of semiconductors^22^. Hence, the device underlying the memory cell is more akin to a high-electron mobility transistor (HEMT) than a MOSFET. The channel is formed by InAs that contains neither any junctions nor doping concentration gradients. It is, however, n-doped to compensate for unintentional background doping and intrinsic Ga vacancies and antisite defects in the underlying GaSb, both of which make the layers naturally p-type^23^. Figure 2a depicts the simulated band alignment together with the electron and hole densities within the layers in the absence of bias. As illustrated in the figure, and well documented in the literature^22^, at the InAs/GaSb interface the InAs conduction band is located below the GaSb valence band, resulting in the flow of electrons from the GaSb into the InAs, and leaving holes in the GaSb. Accumulated electrons/holes can be seen at the InAs/GaSb interface, however, the electrons in the InAs channel are not bound to the InAs/GaSb interface and there is significant electron density throughout the InAs channel. The conductance of the entire channel is dominated by the electrons in the InAs, which will have higher mobility and higher areal density (because of the doping) than the holes in the GaSb. The intrinsic InAs FG is isolated from the InAs channel by a 15-nm AlSb barrier, while double InAs quantum wells (QWs) with triple AlSb barriers serve as a resonant-tunnelling barrier between the FG and the n-doped InAs CG. Hence, in our devices the electrons stored in the InAs FG are isolated by the anomalously-large conduction-band discontinuity with AlSb, a charge-confinement system that was predicted to have a room-temperature thermally-activated storage time of an extraordinary 10^14^ years^24^.

A crucial aspect of the design and operation of the device is that the two QWs (QW_1_ and QW_2_) in the triple resonant-tunnelling barrier have different thicknesses, *i.e*. confined states with different energies^21^ as illustrated in Fig. 2a. As QW_2_ is thinner than QW_1_, the only available energy-level for electrons in QW_2_ is at a higher energy than the equivalent in QW_1_. Furthermore, the state in QW_1_ is at significantly higher energy than the state in the neighbouring CG region. This obstructs direct electron tunnelling between CG and FG, such that, when the system is unbiased, the barrier to electron passage from CG to FG (or vice versa) is given by the InAs/AlSb conduction-band offset of 2.1 eV, *i.e*. no charge will flow to (or from) the FG. In the same way, the ground and the first excited-states in the FG are located well below the energy states within the QWs. Hence, when no voltage is applied, electrons are confined inside the FG, the triple resonant-tunnelling barrier is opaque to the passage of electrons to or from the FG, and non-volatility is achieved. On the other hand, application of a small voltage to the CG allows tuneable coupling of energy states within the resonant-tunnelling barrier such that electrons pass out of (Fig. 2b) or into (Fig. 2c) the FG as required. In this work, read, write and erase operations were conducted on a number of 10 μm × 10 μm (gate dimension) cells in an electrostatically-shielded dark box at room temperature. All processes, including write and erase, were executed with ≤2.6 V bias, which is about an order of magnitude lower than is needed to fully operate a Flash cell. Erase was performed by application of a CG bias, ${V}_{CG-S}^{E}$, of +2.5 or +2.6 V between the CG and the source, resulting in a “0” state. Figure 2b shows the calculated band alignments resulting from applying an erase voltage of +2.6 V. Under such circumstances, the calculated electron energy level in QW_1_ is lower than the level in QW_2_, while both are below the first excited-state and close to the ground-state energy level in the FG. Furthermore, the calculated electron probability density for the ground state in the FG predicts a high electron accumulation at the interface with the resonant-tunnelling barrier and a decaying tail extended into the first AlSb barrier (on the left). The net effect of an erase is thus an electron flow from the FG to the CG, depleting the FG. Similarly, write used ${V}_{CG-S}^{W}=\,-\,\,{V}_{CG-S}^{E}$ to increase the charge in the FG (a “1” state). Figure 2c shows the calculated band diagram when a CG bias of ${V}_{CG-S}^{W}$ = −2.6 V is used to write the data. In this case, the energy levels in QW_1_ and QW_2_ are nearly coincident, leading to a strong coupling of these states, resonant tunnelling, and electron flow from the CG to the FG, charging the FG.

Due to capacitive coupling, the conductivity of the channel depends on the amount of charge stored in the FG, so data is read by measuring the source-drain current, *I*_*S-D*_, for fixed source-drain voltage, *V*_*S-D*_. A “1” state, defined as increased charge in the FG, reduces the charge in the channel, hence its conductivity. Conversely, a “0” state increases the channel conductivity. The data can be read in the absence of any bias to the CG, but such a voltage would be required to select individual devices (bits) in an array of cells, and should generate an electric field across the resonant-tunnelling barrier that is insufficient to allow the passage of charge into or out of the FG. Applying ~2.5 V between the CG and a common back gate can conveniently achieve this.

### Memory characteristics

**Figure 3:**
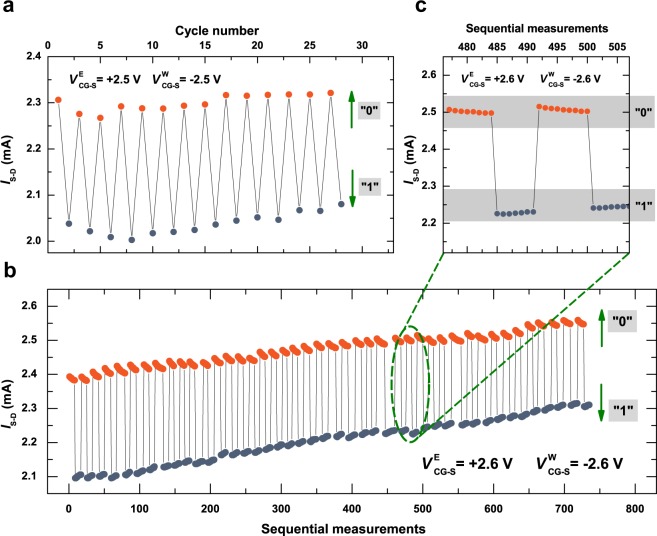
Erase-read-write-read cycles. “0” and “1” states are prepared by application of erase and write voltages, ${V}_{CG-S}^{E}$ and ${V}_{CG-S}^{W}$, respectively, and read via the source-drain current, *I*_*S-D*_, with 1.0 V bias across the channel and zero bias on the control gate. (**a**) Single read measurements subsequent to each erase or write. (**b**) Part of an extended measurement with multiple reads (every 1 s) following each erase or write operation (>80 cycles shown). (**c**) A detail of a portion of the data in **b** showing a slight convergence of *I*_*S-D*_ for “0” and “1” states following erase and write. In (**b**,**c**) the number of reads following each write or erase is arbitrary.

For the results presented here, read was performed with zero bias on the CG, and *V*_*S-D*_ = 1.0 V, although substantially smaller *V*_*S-D*_ can easily be used. Figure 3a shows a series of erase-read-write-read operations, in which a single read follows each write or erase. Part of a substantially extended sequence of erase-read-write-read operations is shown in Fig. 3b, with several reads following each write or erase. This clearly demonstrates the non-destructive nature of the read operation. A clear differentiation between the “0” and “1” states is maintained throughout both sequences, although in Fig. 3b there is an undesirable, but almost symmetric, upward drift in *I*_*S-D*_ as the number of operations increases. The reason for this requires further investigation, but is likely to be an asymmetry in the write-erase process, such that each erase removes slightly more charge from the FG than the write delivers. There is no such drift in Fig. 3a, where the write/erase voltages are slightly lower. Following several hundred write and erase operations and many more reads, in different experiments, there was no sign of damage to the device. Research is currently underway into automated characterisation of endurance of single cell devices, and into defining suitable architectures and operational processes for arrays of memory cells.

In addition to non-volatility and low-voltage write and erase, a low switching energy is an important characteristic of a memory, and one which emerging memories have struggled to compete with DRAM and Flash^25^. In common with those conventional memories, our devices are also based on charge storage, so the switching energy is given by the capacitive charging energy: low-voltage switching is synonymous with low-energy switching. Indeed, since our devices have a similar structure to Flash, simply assuming the same capacitance for the same gate dimension, infers that the switching energy is ~(20/2.5)^2^ = 64 times less than Flash, which also puts it lower than the switching energy for DRAM (for a given device size)^25^. Theoretical evaluations suggest a CG-FG capacitance of the order of 10^−12^ F for a 10 µm × 10 µm (gate dimension) device, and a switching energy of ~2 × 10^−12^ J. Shrinking the device dramatically decreases this number, such that the switching energy is of the order of 10^−17^ J for the 20 nm node, which is 100 and 1000 times smaller than for DRAM and Flash respectively^25^. This potential for ultra-low switching energy in an emerging memory is, to the best of the authors’ knowledge, unique.

### Retention

**Figure 4:**
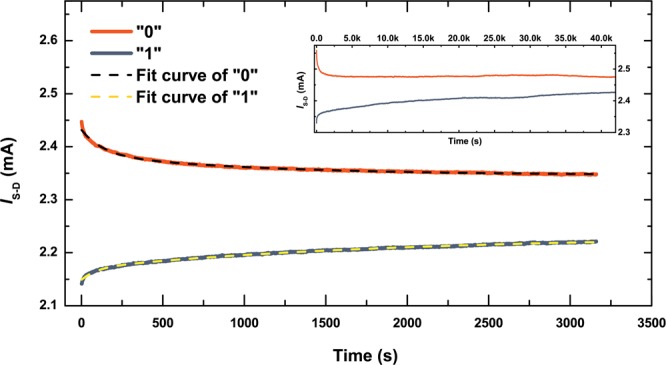
Data retention following write and erase. Time evolution of the “0” and “1” states as measured by the source-drain current, *I*_S-D_, (solid orange and navy lines, respectively) follow double-exponential decay (dashed lines) with asymptotic values that do not converge. Data was taken with 1.0 V source-drain bias and zero bias on the control gate. The inset shows an example of an overnight measurement (>12 hours).

Figure 3c shows a detail of some of the write-erase operations in Fig. 3b, revealing a different variation in the “0” and “1” states: successive read measurements following an erase yield a slightly smaller *I*_*S-D*_ for the “0” state. Similarly, for successive read measurements following a write, *I*_*S-D*_ is slightly larger. This effect is thus different to the upward drift in *I*_*S-D*_ discussed above, and is related to the volatility of the data. To investigate this further we performed successive read operations over extended periods for each memory state. Examples of such measurements are shown in Fig. 4, where the retention characteristics of the memory cells were studied by monitoring their time-dependent behaviour at a constant *V*_*S−D*_ = 1.0 V. For this purpose, a read was performed every second for about an hour following an erase pulse (${V}_{CG-S}^{{\rm{E}}}=+2.6$V, pulse duration 1.0 s). After this experiment, the memory was programmed to the “1” state by a write voltage pulse (${V}_{CG-S}^{{\rm{W}}}=-2.6$V, pulse duration 1.0 s), and the data was read in the same way. Both “0” and “1” states exhibit an initial rapid decay that is consistent with that seen in Fig. 3c. However, for both memory states the initial rapid decay is followed by much slower changes, such that during the entire observation time, the corresponding “0” and “1” states are clearly distinguishable. To further investigate the retention properties of these memory devices, a separate experiment was performed on the same device for an extended period of time. This is illustrated in the inset of Fig. 4, showing the ultimate saturation of the exponential decays and distinct “0” and “1” states over time. Such a storage time is at least 10^6^ times longer than the refresh time required for DRAM.

Fitting the data in Fig. 4 with double exponential decay functions reveals that the initial ‘fast’ component has a time constant of ~100 s, while the time constant for the ‘slow’ component is approximately an order of magnitude longer, for both states. The existence of double exponential decay implies that there are at least two mechanisms behind the degradation of the states. Candidates include tunnelling through defect states in the AlSb barriers, thermal excitation of electrons across the narrow InAs bandgap and recombination with thermally-generated holes. Crucially, the asymptotic values of the “1” and “0” states are 2.232 ± 0.0002 and 2.346 ± 0.0001 mA respectively, indicating that discernible “1” and “0” states could endure indefinitely, *i.e*. the data in Fig. 4 are consistent with non-volatility. We note that even though these states are clearly distinguishable in this experiment, a larger contrast is required for implementation in a practical device. From consideration of the capacitance of the device and the applied write/erase voltage, we estimate that write and erase transfer ~10^7^ electrons to or from the FG. Despite the fact that this is a large number, it has a small effect on the conductivity of the laterally-junctionless InAs channel. This is mainly due to the Fermi-level pinning above the conduction band of the highly doped InAs channel which makes it always conductive and very hard to deplete. Furthermore, the InAs channel is 5 times thicker than the FG, leading to a higher level of intrinsic or thermally-generated carriers in this layer. This can be resolved by making the channel thin and narrow enough to allow for full depletion of carriers when the memory is in the “1” state. Strong quantum confinement and, thus, heavy quantisation is expected at sub-20-nm thicknesses^26,27^.

## Conclusions

We have demonstrated room-temperature operation of non-volatile, charge-based memory cells with compact design, low-voltage write and erase and non-destructive read. The contradictory requirements of non-volatility and low-voltage switching, are achieved by exploiting the quantum-mechanical properties of an asymmetric triple resonant-tunnelling barrier. The compact configuration and junctionless channel with uniform doping suggest good prospects for device scaling, whilst the low-voltages, non-volatility and non-destructive read will minimise the peripheral circuity required in a complete memory chip. These devices thus represent a promising new emerging memory concept.

## Methods

### Simulations

The nextnano^28,29^ software package was utilised for mathematically modelling the energy band diagram of the memory device structure reported here, taking into account strain and piezoelectricity. Within this work, a self-consistent Schrödinger solver was used along with the Poisson and drift–diffusion equations to calculate the electron densities at equilibrium and under bias.

### Molecular beam epitaxy

The InAs/AlSb/GaSb heterostructures were grown on a semi-insulating, undoped, 2-inch GaAs wafers using molecular beam epitaxy (MBE). A GaAs substrate was chosen because of the long-term need for low-cost manufacture, even though the resulting mismatched epitaxy reduced device yield in the present work. First, the (100) GaAs substrate was annealed for 15 minutes at 600 °C to remove the native oxide layer. After oxide desorption, the substrate was cooled down to 570 °C and the growth initiated with a 50-nm GaAs buffer layer followed by a 250-nm-thick n-type (5 × 10^17^ cm^−3^ Si-doped) GaAs layer. This doped layer forms a buried back-gate, but was not used in the experiments reported here. A SUMO® K-cell was used as the Ga source, along with a valved cracker source configured for As dimers. Next, a 100-nm Al_0.8_Ga_0.2_As layer was grown with an Al SUMO® K-cell at the same substrate temperature to separate the GaAs back-gate from the other active layers, in both the processing and electrical measurements of devices. After the growth of a further GaAs buffer layer of 10 nm, the As source was shut for 1 minute to minimise the As background, followed by a 10 s growth interrupt to desorb the As surface layer from the sample. This was in preparation for the growth of the GaSb via the interface misfit method^30^, needed for the transition to the ~6.1 Å lattice constant of the active layers of the device. Following the initial introduction of Sb_2_ flux from a valved cracker source, the substrate temperature was decreased to 500 °C and a 630-nm-thick GaSb layer was grown. The substrate temperature was further decreased to 440 °C for the growth of the n-type InAs channel (5 × 10^17^ cm^−3^ Si doped). Due to the use of radiative heating in the MBE reactor and an undoped substrate, infrared absorption within the narrow-bandgap epilayer led to an increase in surface temperature to an estimated 470 °C during the growth of the InAs channel. The 15-nm-thick AlSb charge-blocking-barrier layer, the 12-nm-thick InAs floating gate and the AlSb/InAs resonant-tunnelling barrier were grown with InSb-like interfaces to ensure abrupt boundaries between materials and good electrical characteristics^31,32^. Finally, the control gate was grown with a 7-nm-thick cap layer of intrinsic InAs followed by 55 nm of heavily-doped (1 × 10^18^ cm^−3^ Si) n-type InAs.

### Device processing

A top-down processing procedure was employed to fabricate the memory devices with gate dimension of 10 μm × 10 μm. In this device design, there are four different electronic terminals: the GaAs back-gate, the source and drain terminals on the InAs channel, and the control gate on the top InAs layer. Separate UV lithography stages were used to pattern the back gate, device mesa and source/drain areas. At each step, excess material was dry etched with an Oxford Instruments Plasma Lab 100 inductively-coupled plasma (ICP) machine. The etching process was carried out using a BCl_3_/Cl_2_/Ar^33,34^(8.5/2.5/5 sccm) gas mixture and a chamber pressure of 4 mTorr, with an ICP power of 120 W and an RF power of 25 W. During the process, the sample holder temperature was kept at 10 °C by the combination of helium flow and the use of FOMBLIN vacuum oil to achieve the desired thermal conductivity between the substrate and the sample holder. After sculpting the surface of the grown wafer, a 200-nm SiO_2_ layer was deposited on the sample using an Oxford Instruments plasma-enhanced chemical vapour deposition machine. The SiO_2_ layer provides device isolation, and prevents any shorts between the subsequent metal contacts in the probing areas and underside layers. In order to attain good adhesion between the hydrophobic SiO_2_ layer and the photoresist, a thin discontinuous layer of Al_2_O_3_ was thermally grown on the surface of the sample. However, to get access to the top and back gates, the source and the drain, the oxide layer was chemically etched (using buffered oxide etch, 10:1) in the contact areas, which were exposed through openings in the photoresist provided by UV lithography. A hard-baked positive photoresist lifting layer was then patterned on one edge of the devices by UV lithography. This enabled deposition of continuous contacts, and at the same time, prevented any shorts between the metal and the layers (other than the desired ones) in regions where the oxide was possibly partly over-etched. To form ohmic contacts to InAs (control gate, source and drain) and GaAs (back gate), saturated ammonium polysulphide ((NH_4_)_2_S:S_x_) was used to remove the native oxide with minimal semiconductor material removal and to passivate the semiconductor surface in the contact areas^35^. Finally, the metal contacts were formed by deposition of 20/100 nm Ti/Au with an e-beam evaporator.

### Electrical measurements

Current-voltage measurements were carried out on individual devices using a dual-channel Keithley 2634B source meter unit. Memory chips were mounted on to a ceramic chip holder, with connections formed by gold wire attached between bond pads and the chip holder. Semi-automated measurements using custom-made software were performed in darkness at room temperature and pressure, with the sample in an electrically-shielded switching box. All measurements reported here were on 10 μm × 10 μm devices. Erase was performed with ${V}_{CG-S}^{E}$ of +2.5 or +2.6 V applied between the control gate and the channel, with source and drain grounded, and similarly for write, with ${V}_{CG-S}^{W}=\,-\,\,{V}_{CG-S}^{E}$. Read was performed by measuring *I*_*S-D*_ with *V*_*S-D*_ = 1 V and the control gate grounded. Write and erase pulses were 1 s in duration, while read pulses were 10 ms. The back gate was not used, and remained grounded. No bias was required to retain the charge in the floating gate.

### Transmission electron microscopy measurements

A conventional method was used to prepare the specimen in cross section for the analysis by HAADF-STEM. This consisted of mechanical thinning by SiC sandpaper to a thickness of 30 µm, followed by Ar^+^ milling using a precision ion-polishing system to create a hole. The area suitable for study is located at the edges of the hole. The beam energy during milling has to be controlled carefully to obtain a high-quality electron-transparent sample; 3.5 kV was used at the beginning, and 2 kV at the last step, to get a clean and smooth surface. The HAADF-STEM study was carried out using a JEOL 2010 F at 200 kV.

## Data Availability

Reprints and permissions information is available at www.nature.com/reprints. The datasets used in generating the figures are available at 10.17635/lancaster/researchdata/275.
